# Artificial intelligence accuracy assessment in NO_2_ concentration forecasting of metropolises air

**DOI:** 10.1038/s41598-021-81455-6

**Published:** 2021-01-19

**Authors:** Seyedeh Reyhaneh Shams, Ali Jahani, Saba Kalantary, Mazaher Moeinaddini, Nematollah Khorasani

**Affiliations:** 1 Department of Environmental Pollution, Faculty of Environment, College of Environment, Karaj, Iran; 2 Research Center of Environment and Sustainable Development and College of Environment, Tehran, Iran; 3 Department of Occupational Health Engineering, School of Public Health, Tehran University of Medical Sciences, Tehran, Iran; 4 Department of Environment, Faculty of Natural Resources, Tehran University, Karaj, Iran

**Keywords:** Environmental sciences, Mathematics and computing

## Abstract

Air quality has been the main concern worldwide and Nitrous oxide (NO_2_) is one of the pollutants that have a significant effect on human health and environment. This study was conducted to compare the regression analysis and neural network model for predicting NO_2_ pollutants in the air of Tehran metropolis. Data has been collected during a year in the urban area of Tehran and was analyzed using multi-linear regression (MLR) and multilayer perceptron (MLP) neural networks. Meteorological parameters, urban traffic data, urban green space information, and time parameters are applied as input to forecast the daily concentration of NO_2_ in the air. The results demonstrate that artificial neural network modeling (R^2^ = 0.89, RMSE = 0.32) results in more accurate predictions than MLR analysis (R^2^ = 0.81, RMSE = 13.151). According to the result of sensitivity analysis of the model, the value of park area, the average of green space area and one-day time delay are the crucial parameters influencing NO_2_ concentration of air. Artificial neural network models could be a powerful, effective and suitable tool for analysis and modeling complex and non-linear relation of environmental variables such as ability in forecasting air pollution. Green spaces establishment has a significant role in NO_2_ reduction even more than traffic volume.

## Introduction

High levels of air pollution is presently one of the significant environmental issues and problems in many countries. Pollutants are a mixture of natural and human-made sources and many of them pass over the standard limit^1,2^. Air pollutants emission has had a considerable influence on the increase in mortality rates, so healthy air is one of the main issues for human and environment^3,4^. Nitrogen dioxide (NO_2_) is one of the important families of air-polluting chemical compounds worldwide. NO_2_ gas is emitted from indoor sources (gas stoves, vented gas heaters, and smoking) and outdoor sources (high-temperature combustion process, mobile sources such as vehicles and traffic, and industrial processes)^5,6^. Moreover, a mainly part of NO2 concentration linked to secondary production of NO_2_ in air through photochemical processes. Most nitrogen oxides are NO, but this gas is rapidly oxidized to NO_2_ in the presence of O_3_ ozone^7^. NO_2_ could lead to many unfavorable impacts on human health, environment and biological ecosystems such as acid rain, ozone layer reduction, and global warming^8^. Epidemiological studies show that high-level exposure to NO_2_ in the air as a pollutant lead to increasing approximately 5–7% of the lung cancers among people (ex-smokers and non-smokers)^9^. Moreover, these studies reveal that a positive correlation between air pollutants such as NO_2_ and adverse effects on human health such as changes in the kidney, liver, heart, and decrease resistance immunity to infectious diseases^10–12^. World Organization Health (WHO) reported that NO_2_ associated with adverse effects such as increases in respiratory symptoms, asthma prevalence and incidence, cancer incidence, adverse birth outcomes and mortality^13^. Air pollution is a huge challenge in metropolitans such as Tehran city as the capital of Iran. The overcrowded population of Tehran and the increasing number of vehicles as well as the concentration of industries are causes of air pollution, in the past two decades^14^. It is estimated that, between March of 2016 and 2017, 5% of days are classified as a clean air day, 71% as moderate clean air day, 22% unhealthy air for the sensitive group, and 2% unhealthy air for all people^15^. There are increasing levels of NO_2_ pollution in many countries, especially in metropolises such as Tehran. Therefore forecast, controlling and counteracting NO_2_ is a vital issue in urban management^4^.

Artificial neural networks (ANNs) are mathematical models that can be used for complex and non-linear processes^16,17^. ANNs can simulate the behavior of the human brain^18,19^. In recent years, ANNs have been successfully used for predicting and modeling the concentration of ambient air pollution. Furthermore, ANNs are widely used in short- and long-term applications for forecasting pollutants^20^. Various studies applied the ANNs for pollution prediction without considering a variety of environmental factors such as green space and parks, which is recognized as a shortcoming^4,21–23^. Regression analysis is a traditional statistical technique for model generation. Multi-linear regression (MLR) analysis is an approach to evaluate the relationship between independent and dependent factors. The goal of this investigation is the prediction of the concentration levels of NO_2_ as a factor determining the atmospheric pollution in Tehran city. For this purpose prediction accuracy, MLR and ANN models are selected to be compared. Many environmental variables were used to support model outputs, and finally, we identified the most accurate model for prediction of the concentration levels of NO_2_ in Tehran. The result of model sensitivity analysis was illustrated to prioritize model variables.

## Material and methods

### Data collection

To perform this research, data from the Tehran Air Quality Control Company, Tehran Meteorological Organization, Tehran Transportation and Traffic Organization and urban green space information were collected. The parameters measured in these organizations were included five groups: the concentration of NO_2_, urban traffic parameters (using Tehran traffic cameras data), urban green space information, meteorological data, and time parameters. The urban traffic parameters were including the length of the north–south (y) and east–west (x) cross streets (km), the average number of vehicles at the street intersections, and total number of vehicles at the intersections. The urban green space information were the number of parks, the total area of parks in each district, an average of the distance between each park and NO_2_ monitoring station and park index adjacent to the NO_2_ monitoring station (i.e. the area of the nearest park to the NO_2_ monitoring station divided by the distance of the nearest park form NO_2_ monitoring station) and the area of green spaces in district. Furthermore, meteorological data such as air temperature, rainfall, wind speed and direction, humidity, air pressure, and the length of sunshine per day, as well as time parameters such as 1-day time delay (NO_2_ concentration in the last day), 2-day delay time (NO_2_ concentration in the past two days), the day of the year(1–365), the desired month (1–30), the season (1: Spring-2: Summer-3: Autumn-4: Winter) and the warm and cold seasons (1: hot-2: cold) were considered as main variables affecting the air pollution of the Tehran. Data were collected for 1 year (2015). The stations of NO_2_ monitoring and meteorological stations were located close to each other and in one area, as well as the traffic information was gathered on the streets near the stations.

### Multiple linear regression model (MLR)

Multi-linear regression analysis (MLR) was implemented in the form of stepwise. This model was used to examine the relationship between the daily concentration of NO_2_ of air as dependent data and influential variables as independent data. The most accurate MLR equation was obtained based upon statistical parameters including the highest correlation coefficient, the lowest mean square error root (RMSE) and the number of descriptors in the model (n), and the greater value of the F statistic^2,24,25^. The R statistic parameter shows the accuracy of the regression line and the greater value of R can better fit between observed and predicted values^26,27^.

### Neural network model

In this study, the daily concentration of NO_2_ of air was predicted using an artificial neural network model. The multilayer perceptron (MLP) architecture has been used successfully to model some difficult and diverse problems and nonlinear functions such as air quality forecasting. It is composed of a system of layered and interconnected neurons or nodes, namely, an input, one or more hidden layers, and output layers^15,28,29^. In the current study, logarithmic sigmoid and linear activation functions were examined to optimize the network. The back-propagation (BP) training algorithm is found to be the most common and powerful nonlinear statistical technique in MLP networks^29,30^. All computations were developed in MATLAB R2016b software. In learning process to detection the performance of designed the neural network model, the following statistical indicators such as correlation coefficient (R^2^) (Eq. 1), mean absolute error (MAE) (Eq. 2), mean square error (MSE) (Eq. 3), and root means square error (RMSE) (Eq. 4) were calculated ^31,32^:

1 $${R}^{2}=\frac{\sum_{i=1}^{n}{\left({\widehat{y}}_{i}-{\stackrel{-}{y}}_{i}\right)}^{2}}{\sum_{i=1}^{n}{\left({y}_{i}-{\stackrel{-}{y}}_{i}\right)}^{2}},$$

2 $$MAE=\frac{\sum_{i=1}^{n}\left|{y}_{i}-{\widehat{y}}_{i}\right|}{n},$$

3 $$MSE=\frac{{\sum_{i=1}^{n}\left({y}_{i}-{\widehat{y}}_{i}\right)}^{2}}{n},$$

4 $$RMSE=\sqrt{\frac{{\sum_{i=1}^{n}\left({y}_{i}-{\widehat{y}}_{i}\right)}^{2}}{n},}$$

where ${y}_{i}$ and ${\widehat{y}}_{i}$ are the targets and network outputs, ${\stackrel{-}{y}}_{i}$ is the mean of target values, and n is the number of samples, respectively.

To determine the most affecting factors on the model output, a sensitivity analysis was performed. In this method, each factor was changed in the range of the standard deviation, while other factors were equal to their value of the average. The standard deviation outputs of the model for each factor change were calculated as the sensitivity of the model finding for that factor.

## Results

### The study area

Tehran metropolis, the capital of Iran, with a population of over 8,000,000 individuals, is located in the south of the Alborz mountains and the northern margin of Iran's central desert. Its geographical longitude is 51° 2′ E to 51° 36′ E with an approximate length of 50 km. Its geographical latitude is from 35° 35′ N to 35° 50′ N with an approximate width of 30 km. The altitude of this city at the northern point is about 1800 m and at the southernmost point is about 950 m above sea level. This city has been extended to 730 km^2^^4,33^.

### MLR model

According to the result of the stepwise-multi linear regression analysis, the desired month is from the desired year, length of the north–south (y) and east–west (x) street, wind speed and direction, rainfall, the air temperature, the humidity, the length of sunshine per day, the one-day time delay and the two-day time delay have a significant impact on the predicted daily NO_2_ concentrations in Tehran.

There is a relation between the rate of change of affecting factors and the air NO_2_ concentration in Tehran. The best result for performance stepwise-multi linear regression in predicting the NO_2_ concentration of air set out in Table 1 and Eq. (5).

5 $${NO}_{2}=0.210{X}_{1}-0.947{X}_{2}-0.409{X}_{3}-0.05{X}_{4}-0.851{X}_{5}-2.202{X}_{6}-0.144{X}_{7}-0.072{X}_{8}+0.294{X}_{9}+0.691{X}_{10}+0.212{X}_{11}+10.031.$$

where ${X}_{1}$ is the desired month is from the desired year, ${X}_{2}$ is the length of the east–west (x) street, ${X}_{3}$ is the length of the north–south (y) street, ${X}_{4}$ is wind direction, ${X}_{5}$ is the wind speed, ${X}_{6}$ is the rainfall, ${X}_{7}$ is the air temperature, ${X}_{8}$ is the humidity, ${X}_{9}$ is the length of sunshine per day, ${X}_{10}$ is the 1-day time delay, and ${X}_{11}$ is the 2-day time delay, respectively.

**Table 1:** Multivariate regression model results in forecasting NO_2_ concentration in Tehran city.

R^2^	RMSE	F	Sig
0.816	13.151	605.079	0.000

**Figure 1:**
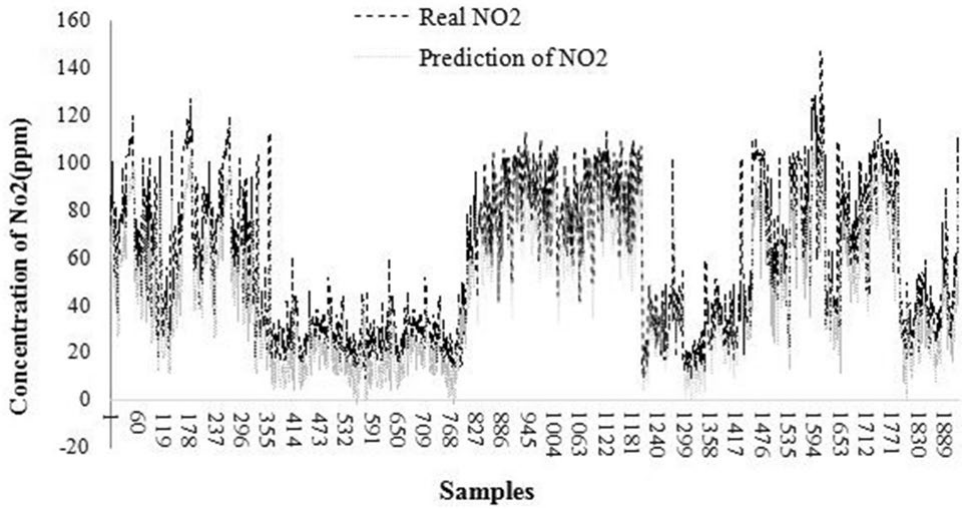
The result of stepwise-multi linear regression in forecasting ting the NO_2_ concentration of air.

The findings obtained from stepwise-multi linear regression in forecasting the NO_2_ concentration of air are shown in Fig. 1.

### ANN modeling

Before neural network training, the factors influencing air pollution, as input data need to be normalized so that the data were converted to numbers between 1 and − 1. Data were randomly divided into three subsets: 20% samples selected for validation, 20% as test sets and 60% as training tests and then input data were weighed in the first layer and moved to the middle layer. Next, the outputs were weighted through connections between the middle layer and the output layer. Finally, the findings were presented in the output layer. The most accurate structure of neural networks optimized using 27 neurons and one hidden layer. The optimal structural characteristics of the neural network are presented in Table 2.

**Table 2:** The optimal structure of an artificial neural network for the forecast of NO_2_ concentration.

Network structural features	Hidden layer	Output layer
Type of network	Multilayer perceptron	Multilayer perceptron
Transfer function	Sigmoid tangent	Linear
Optimal algorithm	Conjugate gradient	Conjugate gradient
Learning period	300	300
Momentum coefficient	0.9	0.9
Number of neurons	26	1
Normalization	− 1 to 1	− 1 to 1

The maximum value of R^2^ as well as the minimum amount of MSE in the test set and train set considered (Table 3). Indeed it shows a very high level of neural network accuracy in forecasting the NO_2_ concentration of air in the city of Tehran.

**Table 3:** The performance of the best artificial neural networks for the forecast of NO_2_ concentration in the air.

Function			Test set				Train set			
activation	Training	Structure	R^2^	MSE	RMSE	MAE	R^2^	MSE	RMSE	MAE
Logsig-Logsig-Purelin	LM	23-26-26-1	0.741	0.235	0.485	0.355	0.879	0.125	0.354	0.252
Tansig-Tansig-Purelin	GDM	23-22-22-1	0.562	0.386	0.621	0.468	0.564	1.631	1.277	0.521
Tansig-Purelin	BQN	23-26-1	0.763	0.208	0.456	0.327	0.836	0.163	0.403	0.278
**Tangsig-Purelin**	**CGB**	**23-26-1**	**0.742**	**0.232**	**0.481**	**0.334**	**0.877**	**0.122**	**0.35**	**0.25**
Tansig-Tansig-Purelin	GDA	23-19-19-1	0.722	0.246	0.496	0.341	0.821	0.181	0.425	0.294
Logsig-Logsig-Purelin	CGP	23-24-24-1	0.711	0.254	0.504	0.353	0.829	0.172	0.414	0.288

*LM* Levenberg–Marquardt, *GDM* gradient descent with momentum, *BQN* BFGS quasi newton, *CGB* conjugate gradient with Powell/Beale, *GDA* gradient descent with adaptive learning rate, *CGP* conjugate gradient with Polak/Ribiere.

**Figure 2:**
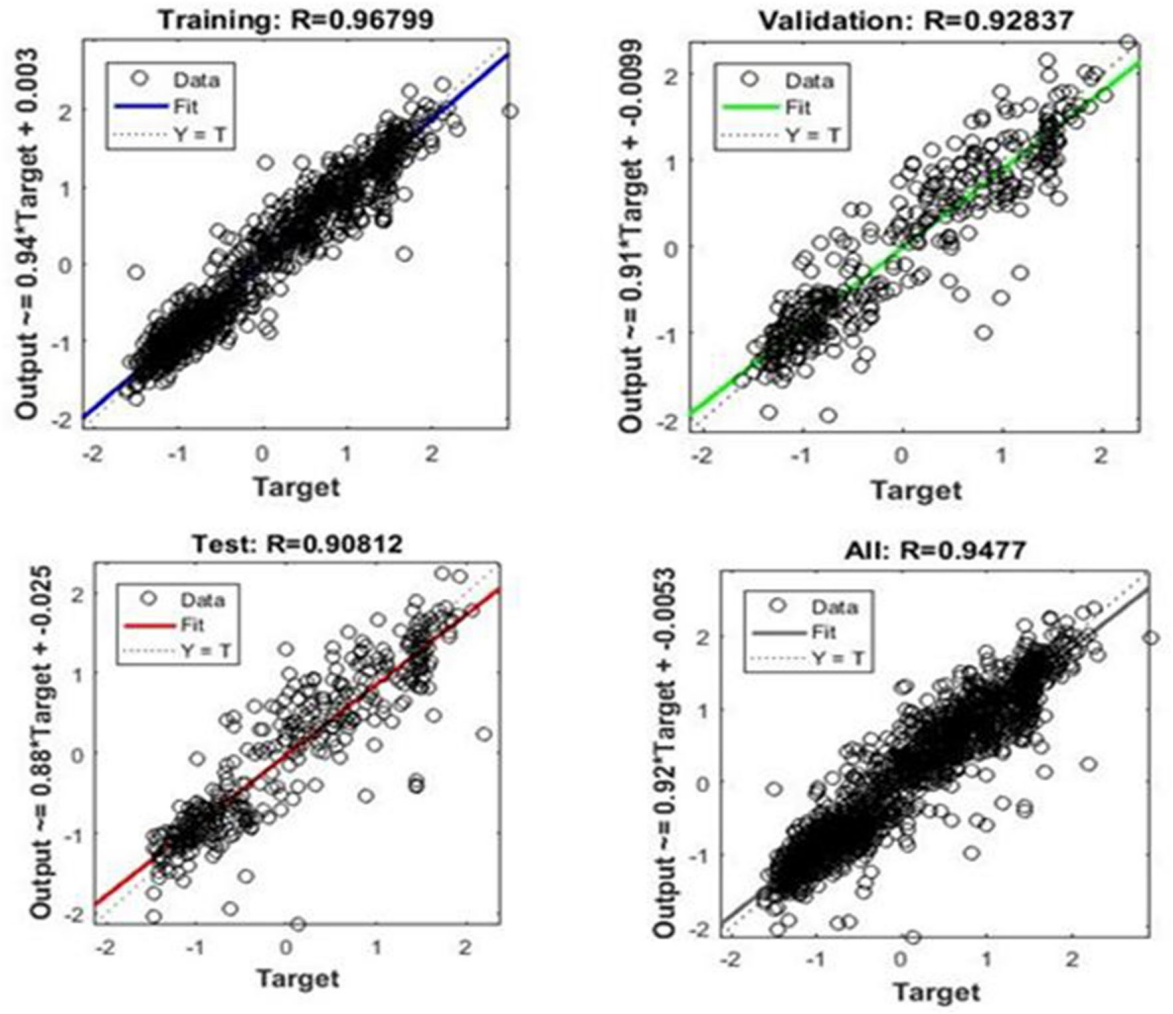
Scatter plots related to data modeling results. Training data, validation data, test data, and total data.

The scatter plot represents a correlation between data that show the accuracy of a neural network model for forecasting the concentration of NO_2_ in Tehran^34^. The scatter plot of the neural network (MLP) output versus target values of the forecasting the NO_2_ concentration of air for training, validation, test, and all data are shown in Fig. 2. As can be seen from Fig. 2, the value of the coefficient (*R*^2^) proves the relatively high correlation between output and target values.

### Sensitivity analysis of NO_2_ concentration

**Figure 3:**
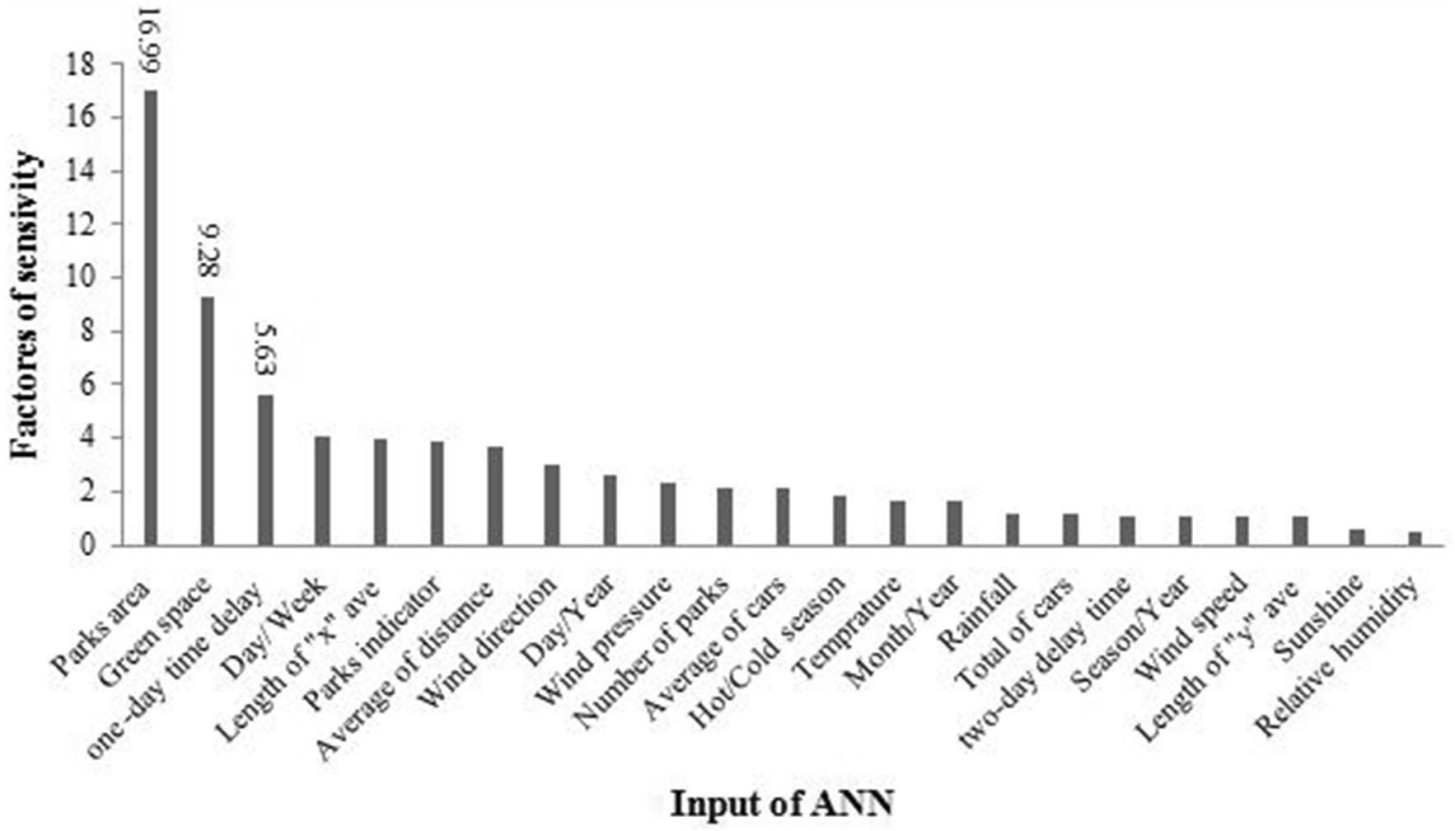
Sensitivity analysis of affecting parameters on NO_2_ prediction.

Sensitivity analysis aims to evaluate the most important input parameters affecting on the model output. As can from Fig. 3, the value of the park area, an average of green space and one-day time delay are detected as the most influential inputs that influence the NO_2_ concentration in the air.

**Figure 4:**
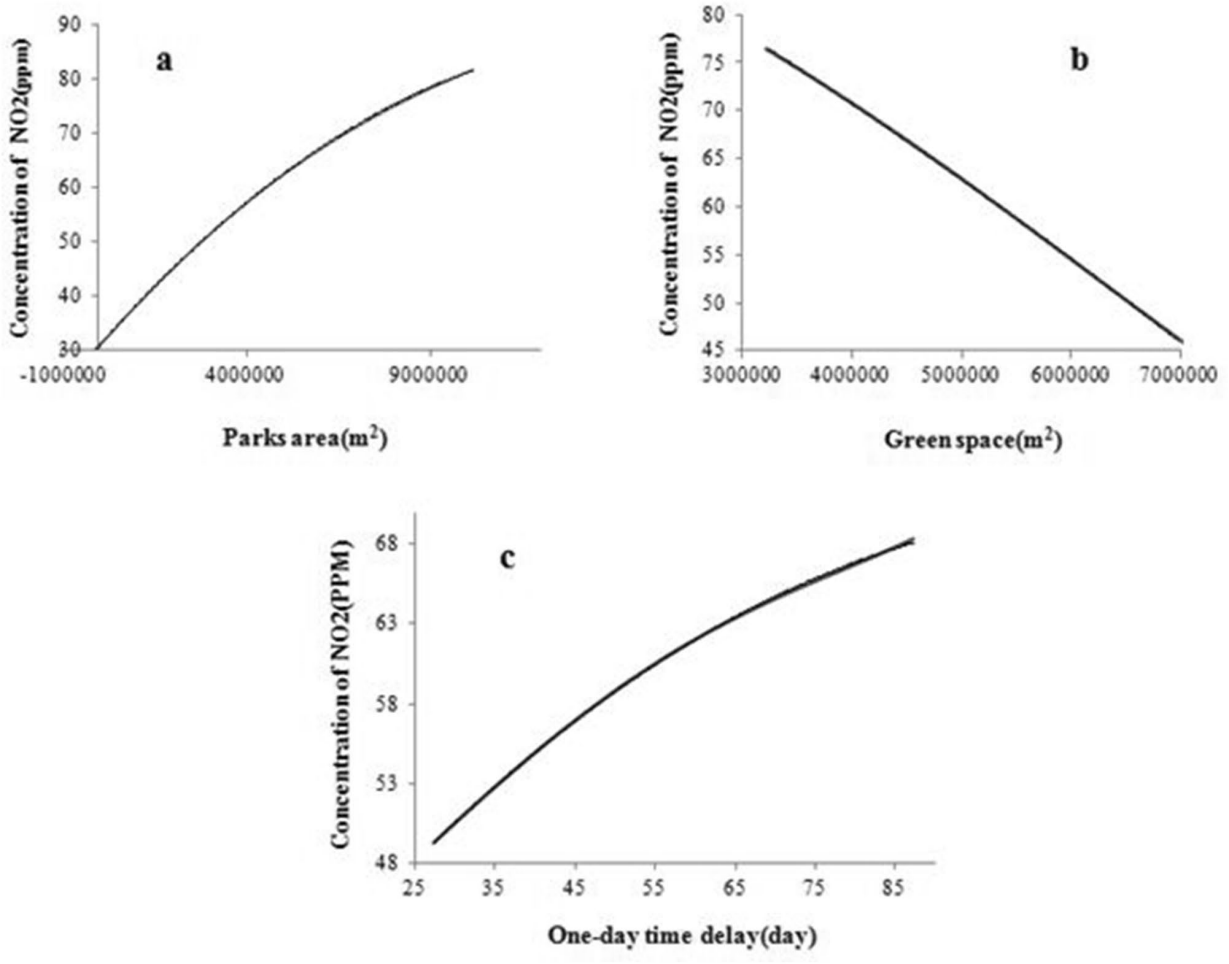
The trend of NO_2_ prediction output changes with varying (**a**) parks area (**b**) green space. (**c**) One-day time delay.

Figure 4 shows the effect of varying most important parameters including park area, area of green space, and one-day time delay on the level of NO_2_ concentration.

## Discussion

The objective of this study was to forecast the air NO_2_ concentration in Tehran metropolis by applying MLR and MLP models. The comparison between MLR and artificial neural network modeling demonstrates that the neural network model (R^2^ = 0.89, RMSE = 0.32) performs more accurately than multiple regression analysis (R^2^ = 0.81, RMSE = 13.151). The MLR analysis had a lower R^2^ value than the MLP model. There is no doubt that air pollution is influenced by several factors including meteorological parameters, pollutant sources, green spaces quality, and quantity, etc. which require a non-linear computing tool as alternatives to the traditional approach. Artificial Neural Network (ANN) models could be used as interpolation methods for complex and non-linear problems such as predicting and modeling air pollution^17,35,36^. Several research exhibits the performance of ANNs and traditional regression models to predict air pollutant concentrations^19,37–39^. Dragomir et al. compared the multiple linear regression and multilayer perceptron for the forecast of the NO_2_ concentration in Romania. This study focuses on the dependence between meteorological factors including temperature, pressure, wind speed, wind direction, solar radiation, rainfall, and relative humidity and their influence on measured NO_2_ concentration. The results indicated that MLP has a higher correlation coefficient than MLR in the forecasting of air quality and meteorological factors have an impact upon the NO_2_ concentration^38^. Rahimi used MLP and MLR for prediction of the NO_2_ and NO_x_ concentrations according to meteorological variables. The best structure for the MLR model RMSE and R^2^ were 3.6 and 0.42 respectively in comparison to the MLP model with RMSE = 0.0046 and R^2^ = 0.82. The findings in this study shows that performance of MLP is superior in comparison to MLR for prediction NO2 concentrations in urban environments^39^. Cabaneros et al. illustrated that MLP models can accurately forecast NO_2_ concentrations (R^2^ = 0.9, RMSE = 23.45) based on air pollution and meteorological data^19^. Cakir and Sita (2020) developed a non-linear method (MLP) and MLR model utilizing air pollution and meteorological data to predict concentration values of NO_2_. The results demonstrate that there are no significant differences between two methods. They recommended that more studies be done in this area^40^. Although there are several studies in this area, however, they did not formulate comprehensive influential variables with green space and traffic data. To determine the daily averaged concentration of PM10, and PM2.5 in the Adriatic coast of Italy, Biancofiore et al. compared ANN and MLR models. This research was used, as input, daily values of air temperature, humidity, air pressure, wind speed, and wind direction. The neural network model has the best correlation coefficient (R = 0.88) and performance compared to the MLR model (R = 0.86) in forecasting air pollution^41^. Alimissis et al. worked on an air pollution prediction model based on artificial neural networks and multiple linear regression in the greater area of metropolitan Athens in Greece. The selected air pollutants were NO_2_, NO, O_3_, CO, and SO_2_. The findings for each five air pollutants highlighted that neural network models to be significantly superior compared to MLR owing to their ability to effectively predict the complex and nonlinear issues such as air pollution^42^. Although implementation of regression methods is simple, however, the results of various studies also exhibit that regression methods may not offer precise predictions in some areas such as air pollution in comparison to ANNs models; and ANNs have generally superior performance^39,43,44^.The comprehensive data collection including green space and traffic data is the most advantages of our study. As a result, our ANN model is more accurate than other developed models in research. It is concluded that green space and traffic variables, especially in metropolitans, should be considered to achieve more accurate models.

As can from Fig. 3, green spaces and parks have a key role in the prediction of NO_2_ air. Figure 4A presents that there is a positive correlation between the concentration of NO_2_ and the park^’^s area. A reason for this result could be the fact that the vegetation cover of parks especially in metropolises are so limited to grasses and bushes and lack of trees is one of the disadvantages. Hence, more human activities around urban parks cause more air pollution. On the other hand, urban green spaces (vegetation in parks, squares, street tree lines, etc.) play a significant role in NO_2_ reduction in the air. This observation is in agreement with those of the previous studies^45–50^. Slemi et al. provided that tree cover and the level of air pollutant concentrations were two key parameters in removing pollutants^47^. Janhall showed that differently designed vegetation and vegetation cover dispersion impact on air quality. Moreover, trees and vegetation should be high and porous enough to allow air to pass through them because the air that passes not through vegetation is not filtered; and vegetation should be as close to sources of contaminants as possible^45^. Vos et al. indicated that trees could result in a higher concentration of NO_2_. This result may be explained by the fact that vegetation cover may lead to obstructing the wind flow. This means that the aerodynamic effect decreases ventilation and filtering capacity of vegetation^51^. As one can see Fig. 4B, there is a negative correlation between the concentration of NO_2_ and area green space, so the concentration of NO_2_ decreases with the increased area green space. Selmi et al. demonstrate the contribution of trees in public green spaces in air pollution removal in Strasbourg city, France. The results showed that trees removed about 88 tons of pollutants during a year. The amount of air pollution removed by trees was approximately 14 tons for NO_2_, 1 ton for CO, 56 tons for O_3_, 12 tons for PM10, 5 tons for PM2.5, and 1 ton for SO_2_^48^. In the United States, urban vegetation is estimated to occupy 3.5% of the land, which could lead to the absorption of 0.711 ton of pollutant. This amount is equivalent to 3.8 billion US dollars. Urban green spaces are often regarded as a substantial air purification service^52^. Although, the tree cover and the level of air pollutant concentrations were two key parameters in removing pollutants^47^. Moreover, we concluded that green spaces has a significant role in NO_2_ reduction even more that traffic variables.

## Conclusion

Atmospheric pollution is now recognized as a permanent concern and a major environmental issue at the global level with adverse health worldwide. Because air pollution can effect on human health, quality of life and environment, the predicting of air pollution values has received much attention number of researchers due to closely relate it health of people and environment. The results showed an accurate prediction approach from the field of data processing techniques and artificial neural network model could be a powerful, effective and suitable tool for analysis and modeling complex and non-linear relation of environmental variables such as ability in forecasting air pollution. Green spaces establishment has a significant role in NO_2_ reduction even more than traffic volume. It can thus be suggested developing different ANNs techniques survey for ambient air pollutants prediction and forecasting. It’s worthwhile in the aspect of environmental, health, economic aims and useful for governmental authorities and decision-makers.

## Data Availability

The data that support the findings of this study are available from the corresponding author upon reasonable request.
